# Diffuse large B‐cell lymphoma presenting with pulmonary artery compression symptoms, case reports

**DOI:** 10.1002/rcr2.1441

**Published:** 2024-08-11

**Authors:** Mhd Baraa Habib, Khaled Ali, Mohamad Fael, Bana Sabbagh, Mohamad Talal Basrak, Osama Alkhalaila, Awni Alshurafa

**Affiliations:** ^1^ Internal Medicine Department Hamad Medical Corporation Doha Qatar; ^2^ Community Medicine Department Hamad Medical Corporation Doha Qatar; ^3^ Faculty of Medicine Damascus University Damascus Syria; ^4^ Emergency Medicine Hamad Medical Corporation Doha Qatar; ^5^ Department of Cardiology Hamad Medical Corporation Doha Qatar; ^6^ Department of Hematology National Center for Cancer Care and Research, Hamad Medical Corporation Doha Qatar

**Keywords:** large vessels compression, lymphoma, mediastinal

## Abstract

Mediastinal diffuse large B‐cell lymphoma (DLBC) may manifest in different presentations including systemic symptoms and local mass symptoms. We report two cases of diffuse large B‐cell lymphoma presenting with pulmonary artery compression symptoms. The first case is of a 38‐year‐old Asian man which mimicked pulmonary embolism, and the second one is of a 27‐year‐old Asian woman who presented with fainting and respiratory symptoms due to local mass effect. Both cases were significantly improved after treatment. Local mass effect symptoms may be the first clinical presentation in DLBC lymphoma and should not be overlooked.

## Graphical abstract

Mediastinal diffuse large B‐cell lymphoma (DLBC) may manifest in different presentations including systemic symptoms and local mass symptoms. We report two cases of diffuse large B‐cell lymphoma presenting with pulmonary artery compression symptoms. Local mass effect symptoms may be the first clinical presentation in DLBC lymphoma and should not be overlooked.

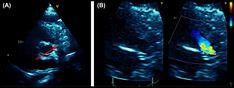

## INTRODUCTION

Accounting for approximately 30% of all non‐Hodgkin lymphoma cases, diffuse large B‐cell lymphoma (DLBCL) is the most prevalent subtype, and it presents with a wide range of symptoms, depending on the location and extent of the disease.^1^ Although can present as a mass anywhere in the body, the most frequent locations for DLBCL are the neck and abdomen, with extra nodal manifestations happen to be found more frequently in stages 1 and 2 of the disease.^2^ Moreover, around one‐third of patients experience systemic B symptoms, especially fever, night sweats, and weight loss.^3^, ^4^

Mediastinal involvement is considered uncommon, with only 7% of diffuse large B cell lymphomas arise primarily from the mediastinum.^4^ However, it is considered an oncologic emergency as it may cause serious complications such as airway compromise and superior vena cava (SVC) syndrome.^5^, ^6^ Lungs, pleura, and pericardium may be involved, causing pleural or pericardial effusion in around 50% of patients. Consequently, respiratory symptoms related to local invasion such as dyspnea and cough are frequently present.^7^ It is difficult to differentiate between systemic DLBCL secondarily involving the mediastinum, and primary mediastinal (thymic) large B cell lymphoma. Yet, bone marrow involvement and Immunohistochemistry can help in making a definite diagnosis.^8^

We present two rare, unique, and unusual cases of DLBCL presenting with great vessels compression symptoms. The first case mimicked pulmonary embolism and the second presented with fainting. It raises the importance of maintaining a broad differential diagnosis in patients with unexplained symptoms, and the need for prompt and thorough investigations to ensure timely and appropriate management.

## CASE REPORTS

### Case 1

A 38‐year‐old man with no chronic illness presented to the emergency department with acute left‐sided chest pain for about 30 min, associated with shortness of breath, vomiting, and on‐and‐off epigastric pain. The patient also reported a weight loss of about 7 kg over the last month. Upon presentation, the patient was afebrile, tachycardic (122 bpm), and tachypneic (30 br/min). Blood pressure was 110/90 mmHg and oxygen saturation was 91% on room air. Chest examination showed decreased breathing sounds at the left lower zone. On palpation, there was a 2 cm mass on the anterior chest at the 3rd intercostal space of the left sternal border. The mass was painless, fixed, round, and had regular borders. The rest of the examination was normal.

**FIGURE 1:**
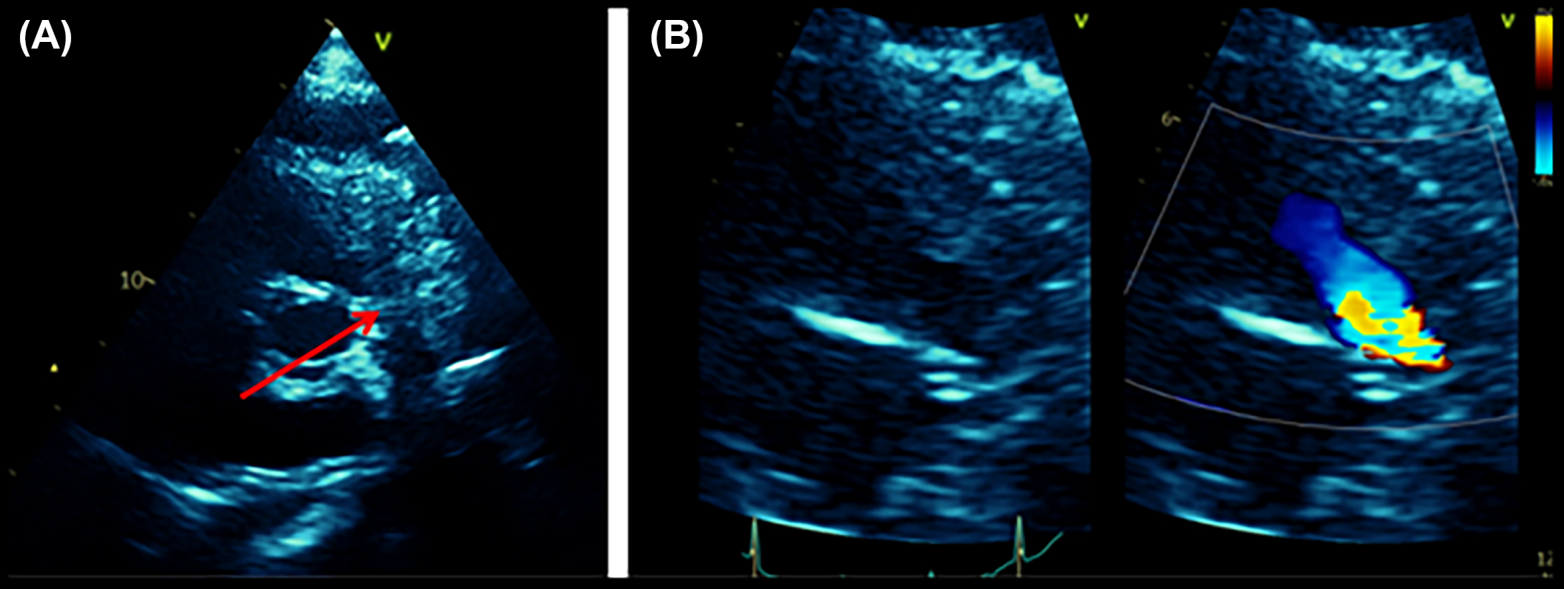
(A) Transthoracic echocardiography (TTE) parasternal short‐axis view at the great vessels level shows compression of the main pulmonary artery (mPA) (red arrow) by extra‐cardiac ill‐defined heterogeneous hyperechoic mass. (B) A focused view of the right ventricular outflow tract (RVOT) and mPA with colour Doppler shows aliasing in mPA suggestive of significant narrowing.

**FIGURE 2:**
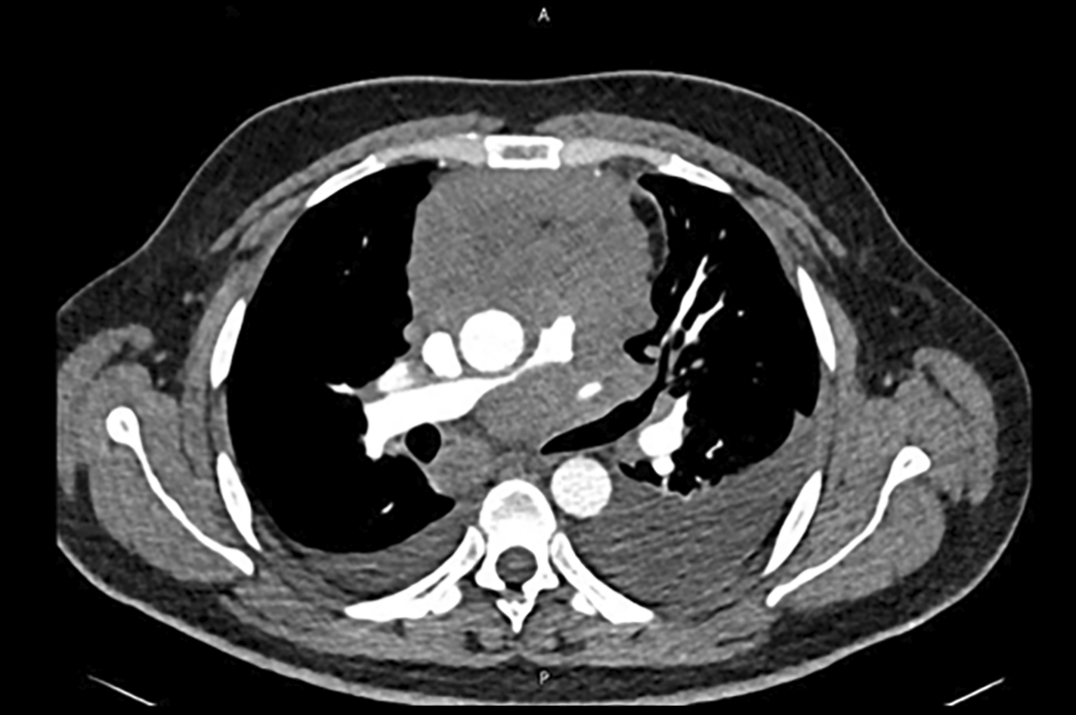
There is a large soft tissue mass in the mediastinum measuring about 9 × 8 cm extending from the thoracic inlet downwards. The mass is seen to encase the main pulmonary artery and its right and left branches.

Blood tests were significant for high LDH (957 U/L), D‐Dimer (1.59 mg/L) and Pro‐BNP levels (3215 pg/mL). The electrocardiogram showed sinus tachycardia with nonspecific T waves inversion. Urgent echocardiography showed a severely dilated right ventricle and severely reduced right ventricular function. The main pulmonary artery appeared compressed. Pulmonary artery pressure couldn't be accurately assessed by echocardiography due to absence of tricuspid regurgitation (TR) jet (Figure 1). These findings were suggestive of pulmonary embolism, so CT pulmonary angiogram was performed. It excluded filling defects within the pulmonary artery divisions; however, it revealed a large mediastinal mass, mediastinal lymphadenopathy, pleural and pulmonary nodules, and bilateral pleural effusion (Figure 2).

CT‐guided mediastinal mass biopsy was performed. Microscopic examination, immunochemical stains, and flow cytometry were diagnostic for diffuse large B‐cell lymphoma, predominantly germinal center type. The malignancy was a triple protein expression of cellular myelocytomatosis oncogene C‐MYC, B‐cell leukaemia/lymphoma 2 BCL‐2, and B‐cell lymphoma 6 BCL‐6).

**FIGURE 3:**
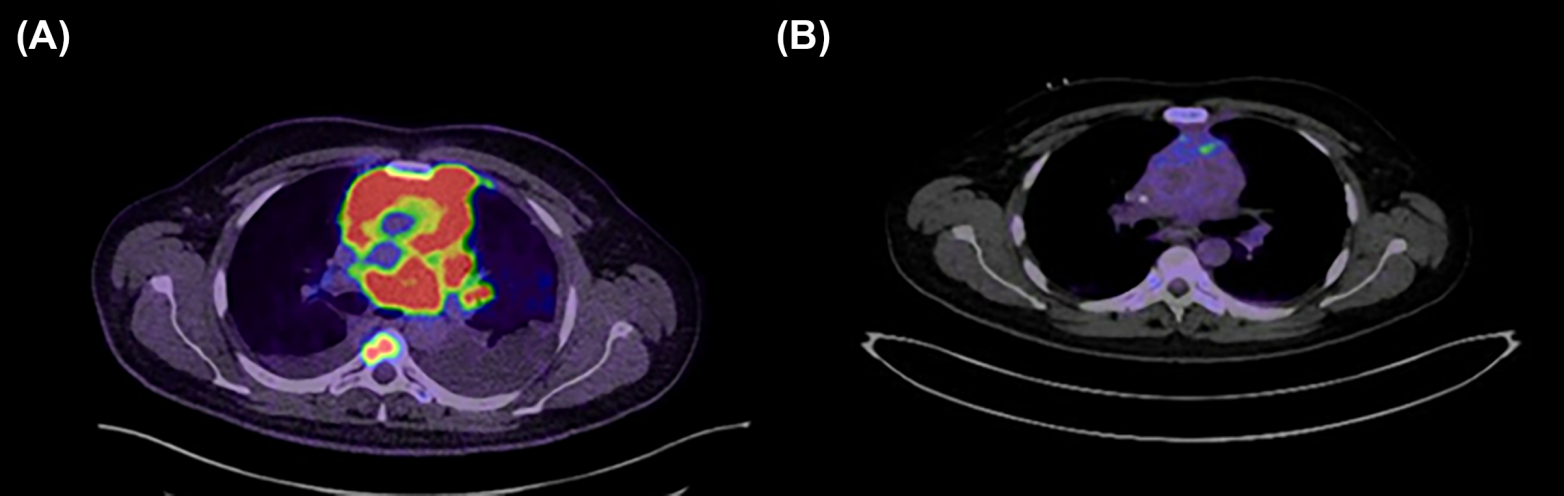
(A) An intense FDG avid mass was seen in the anterior mediastinum extending into the anterior chest wall and posteriorly to the mid/posterior mediastinum region. (B) Interval almost complete resolution of the intense FDG uptake that was previously seen mediastinal region, lower bilateral paratracheal, subcarinal, left hilar and right posterior mediastinum.

Bone marrow biopsy was normal with no evidence of bone marrow involvement by B‐cell neoplasm. The viral tests for CMV, EBV, and HIV were negative. Whole‐body fluorodeoxyglucose‐positron emission tomography FDG‐PET CT was ordered, and it demonstrated extensive hypermetabolic malignant disease features, likely high‐grade lymphoma along with Multiple hypermetabolic lymph nodes seen above and below the diaphragm with multiple FDG avid lesions in different locations (Figure 3A). The patient was diagnosed with DLBL stage 4‐E, CNS IPI score 6 (adrenal–renal involvement).

The patient was started on high‐flow nasal cannula with FiO_2_ of 60% and prophase chemotherapy of cyclophosphamide, oncovin/vincristine, and prednisone (COP), along with rasburicase and a high rate of IV fluid to avoid tumour lysis syndrome. The patient started to improve gradually by day five and was wholly weaned off oxygen by day 10. After discussing the case in a multidisciplinary meeting, we agreed to start him on an R‐CODOX‐M‐IVAC regimen, which consists of Rituximab, Doxorubicin, Vincristine, Cyclophosphamide, Cytarabine, Methotrexate, Etoposide, ifosfamide and Mesna. An echocardiogram one‐month post‐chemotherapy showed a significant improvement in left ventricular function, with normal pulmonary artery pressure, and an interim PET scan after two cycles revealed complete metabolic remission (Figure 3B).

### Case 2

A previously healthy 27‐year‐old woman presented to the ED with an episode of fainting. It was preceded by a three‐month history of non‐productive cough, fatigue, night sweats, and 5 Kg unintentional weight loss. On admission, she was afebrile, blood pressure was 120/75 and heart rate was 80 beats per minute. Physical examination revealed decreased breathing sounds on the left lower lung zone, with no palpable lymph node or organomegaly.

**FIGURE 4:**
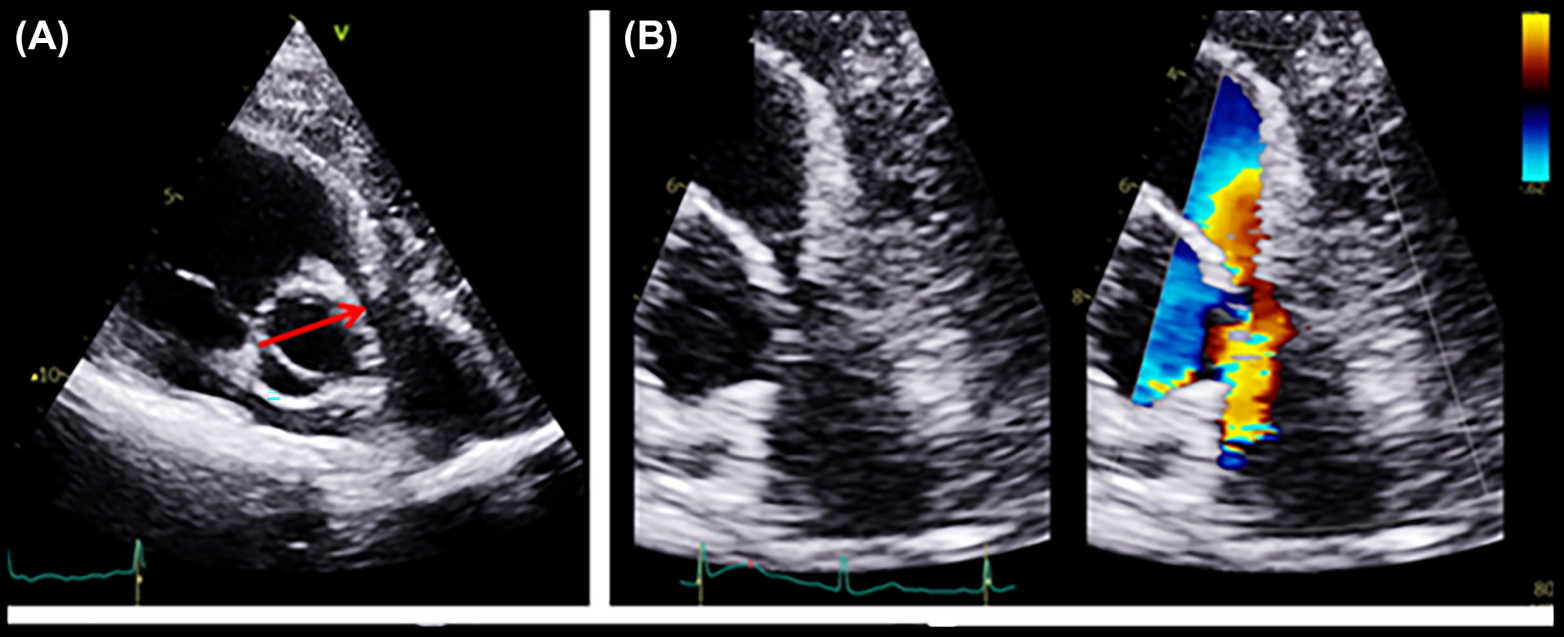
(A) Transthoracic echocardiography (TTE) parasternal short‐axis view at the great vessels level shows compression of the main pulmonary artery (mPA) (red arrow) by extra‐cardiac ill‐defined heterogeneous hyperechoic mass. (B) A focused view of the right ventricular outflow tract (RVOT) and mPA with colour Doppler shows aliasing in mPA suggestive of significant narrowing.

**FIGURE 5:**
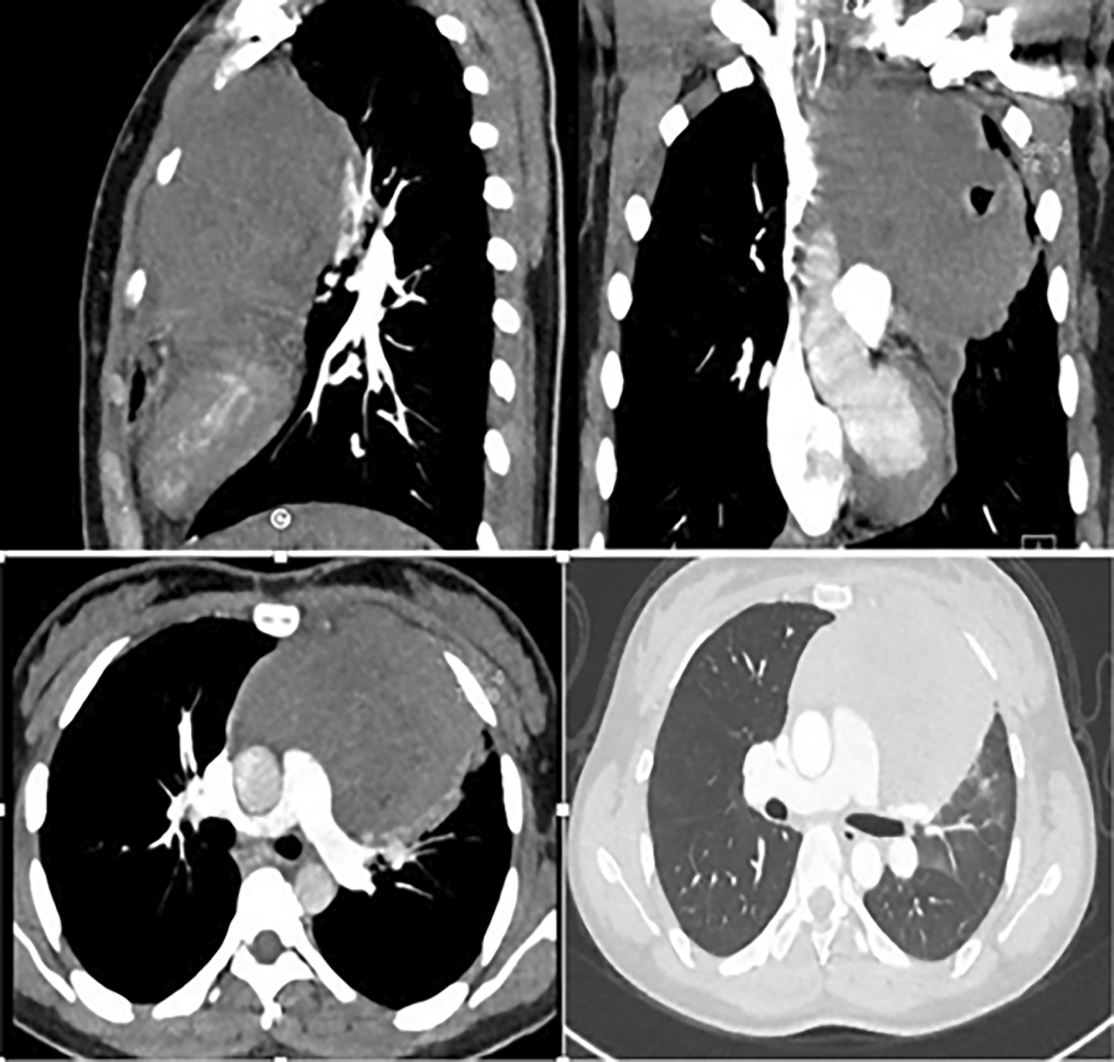
Large midline thoracic lesion with cavitation likely originating from the mediastinum extending to the left upper lung lobe and left chest wall with preserved aerodigestive tract.

Laboratory tests showed for mild microcytic anaemia (Hb 11.7 gm/dL), mildly elevated D‐Dimer (0.58 mg/L) and high LDH (589 U/L). ECG was unremarkable. Urgent Echocardiography revealed normal LV and RV function; however, there was evidence of external pulmonary artery compression (Figure 4). CT pulmonary angiography showed no evidence of pulmonary embolism; however, it revealed a large mediastinal mass (6.8 × 9.7 × 10 cm) (Figure 5). Core biopsy was done and showed diffuse lymphoid blasts infiltration consistent with diffuse large B cell lymphoma with CD23 expression. MYC and CD21 were negative. HIV, EBV, CMV were negative.

**FIGURE 6:**
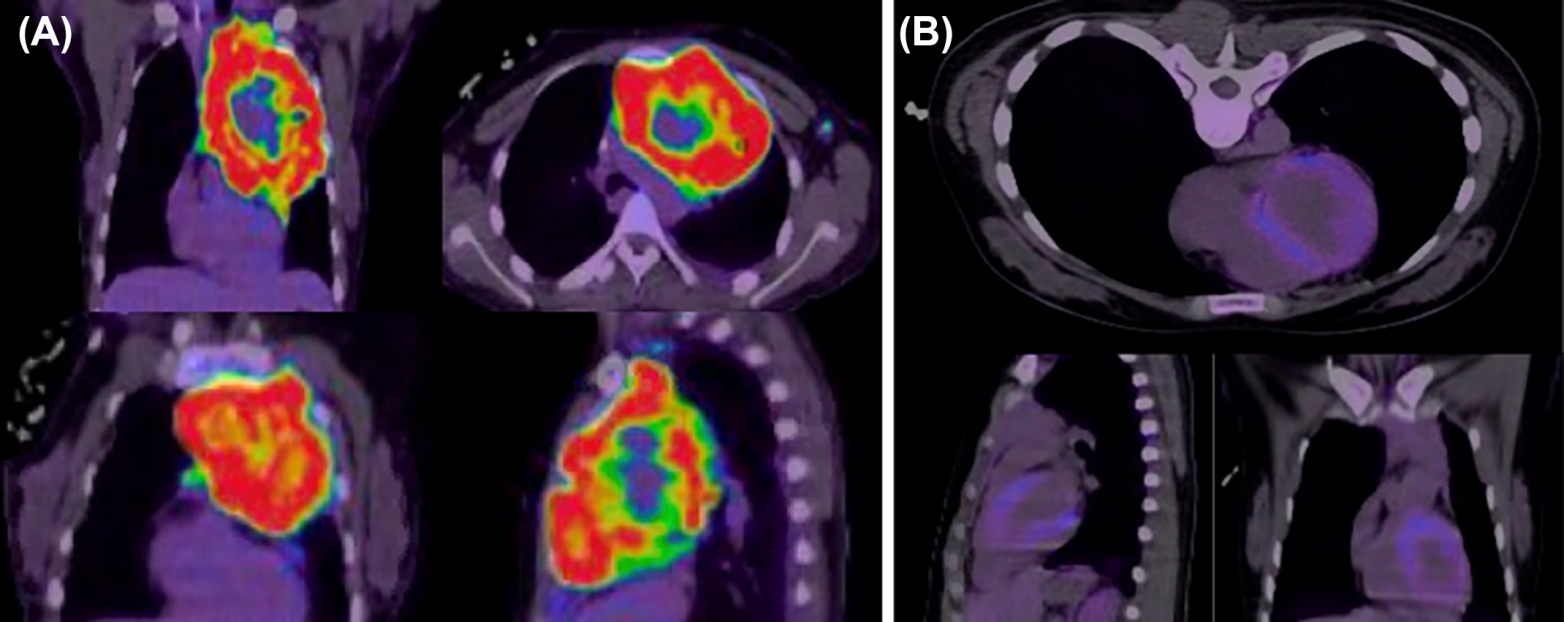
(A): NM FDG PET CT scan showing mediastinal mass consistent with lymphoma. (B): NM FDG PET CT scan showing mediastinal mass consistent with near complete metabolic resolution of the previously noted anterior mediastinal FDG avid mass lesion.

Later, whole‐body FDG‐PET CT scan showed Intense uptake in the mediastinal mass, with involvement of pericardial, left pleural, and bone (T3) (Figure 6A). The patient was considered as Diffuse large B‐cell lymphoma stage IV, IPI: 4, and started on a chemotherapy regimen of rituximab, etoposide phosphate, prednisone, vincristine, cyclophosphamide, and doxorubicin hydrochloride(R‐EPOCH), with CNS prophylaxis.Following four cycles of R‐EPOCH, repeated FDG‐PET CT scan revealed significant decrease in size and near complete metabolic resolution of the anterior mediastinal FDG avid mass lesion (Figure 6B).

## DISCUSSION

The clinical manifestations of mediastinal and thoracic malignancies may vary widely, frequently posing a diagnostic challenge due to the diverse symptoms they have. This complexity necessitates a comprehensive understanding to ensure precise diagnosis and effective management strategies. Among the possible complications, pulmonary artery obstruction represents a rare yet serious consequence of lymphomas impacting the mediastinum. Understanding and addressing such complications requires a thorough assessment of the intricate interplay between the tumour and surrounding structures, emphasizing the importance of a multidisciplinary approach in delivering optimal patient care.^9^

Albeit important as the two have different clinical and pathological features, the definite differentiation between systemic DLBC lymphoma and primary mediastinal (thymic) lymphoma is difficult. Our cases were considered as DLBC lymphomas since we did not find any diagnostic characteristics toward primary mediastinal disease, such as CD200, MAL (Myelin and lymphocyte), or TRAF (TNF receptor‐associated factor).^10^, ^11^

Mediastinum involvement can manifest with compression symptoms of large vessels, trachea, and even pericardial tamponade.^6^ On CT, great vessels encasement was found in around 62% of patients.^12^ In our first case, the mass was surrounding the main pulmonary artery and its branches which caused the presenting clinical picture of pulmonary obstruction. It is also evident in the remarkably high level of pro‐BNP. In the second case, although the lesion was larger in size, the vessel compressing symptoms were less prominent, with more of a lung involvement presentation. Pericardial involvement by mediastinal lymphoma, though rare, presents clinically significant challenges, potentially leading to effusion, constriction, or tamponade. Detection through imaging modalities is crucial for timely intervention, with management typically involving a close collaboration among different specialties, essential for optimizing patient outcomes and preventing life‐threatening complications.^13^

Two cases of constriction of the pulmonary artery secondary to mediastinal lymphoma are reported. Both patients had clinical symptoms of pulmonary embolism. Radionuclide lung scans showed no perfusion of the right lung in one patient, and diminished perfusion of the right lung in the other. Pulmonary angiography revealed diffuse concentric narrowing of right main pulmonary arteries in both patients and of lobar branches in one, in whom severe encasement of the right pulmonary artery by a lymphomatous mass confirmed at autopsy.^14^

A reported case of a 37‐year‐old man with pulsus paradoxus, cough, and fatigue was found to have pericardial effusion, pleural effusions, and a large mass in the upper anterior mediastinum. The mass, which was Hodgkin lymphoma, was compressing the right atrium and pulmonary artery. Chemotherapy was initiated and the patient's symptoms resolved with a decrease in the size of the mass.^15^ Also, another case of a patient with high grade non‐Hodgkin's lymphoma experienced progressive exertional dyspnea was reported. Transthoracic echocardiography revealed compression of the right pulmonary artery.^16^

Another article described a patient with an anterior mediastinal tumour and cardiomegaly detected on a plain chest x‐ray. Echocardiographic‐Doppler examination revealed significant pericardial effusion causing hemodynamic compromise and external compression of the right atrium and pulmonary artery. Pericardiocentesis was performed, and a biopsy obtained during mediastinoscopy diagnosed lymphoblastic lymphoma.^17^

A number of mediastinal neoplasms, such as mesothelioma, lipoma, and even benign tumours, may present with similar symptoms, especially pericardial tumours. Patients usually have dyspnea, chest pain, and palpitations. Also, these tumours can compress great vessels causing hemodynamic symptoms and signs.^18^ Primary cardiac lymphoma was reported to involve the great vessels as well and present with similar symptoms.^19^

A review of 35 published reports shows that teratomas and Hodgkin's disease are the most common neoplasms reported to cause extrinsic pulmonic stenosis. Symptoms commonly reported were chest pain and dyspnea. Pulmonic ejection murmur was the most common physical finding in patients with acquired pulmonic stenosis. The article notes that the prognostic significance of acquired pulmonic stenosis secondary to mediastinal tumours is unclear.^20^

Urgent management of lymphoma with a major vascular compromise involves a multidisciplinary approach. Immediate stabilization with oxygen therapy and hemodynamic support may be necessary. Diagnostic imaging, such as CT angiography, is crucial for confirming the diagnosis and assessing the extent of obstruction. Prompt initiation of chemotherapy or targeted therapy is essential to reduce tumour burden and alleviate obstruction. In severe cases, interventions like thrombolytic therapy, percutaneous interventions, or surgical resection may be considered to restore blood flow. Our cases have favourable responses to systemic chemotherapy.^20^, ^21^, ^22^

In conclusion, mediastinal compression symptoms may be the main and first manifestations of diffuse large B cell lymphoma. These symptoms must be thoroughly investigated to make the right diagnosis at the right time.

## AUTHOR CONTRIBUTIONS

**Mhd Baraa Habib**: Conceptualization. **Bana Sabbagh**: Literature review. **Khaled Ali**, **Mohamad Fael**, **Mohamad Talal Basrak**: Writing the first draft. **Osama Alkhalaila**: Preparing figures and legends. **Awni Alshurafa**: Final revision.

## CONFLICT OF INTEREST STATEMENT

None declared.

## ETHICS STATEMENT

Ethical approval is not required for this study in accordance with local or national guidelines. Written informed consent was obtained from the patients for publication of this case report and any accompanying images.

## Data Availability

Data sharing not applicable to this article as no datasets were generated or analysed during the current study.
